# **Algorithm-based intraoperative diagnosis of liver tumors using infrared spectroscopy**

**DOI:** 10.1038/s41598-025-06250-z

**Published:** 2025-06-20

**Authors:** Rimante Bandzeviciute, Grit Preusse, Sascha Brückmann, Alexander Hirle, Anne Wedemann, Franziska Baenke, Marius Distler, Carina Riediger, Jürgen Weitz, Valdas Sablinskas, Justinas Ceponkus, Gerald Steiner, Christian Teske

**Affiliations:** 1 Institute of Chemical Physics, Faculty of Physics, Vilnius University, Vilnius, Lithuania; 2 Department of Anesthesia and Intensive Care, Clinical Sensoring and Monitoring, Faculty of Medicine Carl Gustav Carus, University Hospital, Technische Universität Dresden, Dresden, Germany; 3 Institute of Pathology, University Hospital Carl Gustav Carus, Dresden, Germany; 4 Department of Visceral, Thoracic and Vascular Surgery, Faculty of Medicine and University Hospital Carl Gustav Carus, Technische Universität Dresden, Dresden, Germany; 5 National Center for Tumor Diseases (NCT/UCC), Dresden, Germany: German Cancer Research Center (DKFZ), Heidelberg, Germany; Faculty of Medicine and University Hospital Carl Gustav Carus, Technische Universität Dresden, Dresden, Germany; Helmholtz-Zentrum Dresden - Rossendorf (HZDR), Dresden, Germany

**Keywords:** Biophysical chemistry, Infrared spectroscopy, Surgical oncology

## Abstract

Liver cancer, including hepatocellular carcinoma (HCC), cholangiocellular carcinoma (CCC), and metastases, presents diagnostic challenges during surgery due to its infiltrative nature. Accurate intraoperative classification and margin assessment are crucial for improving outcomes. Current methods, like frozen section analysis, are time-consuming and subjective, necessitating rapid, objective alternatives. This study assessed fiber-based attenuated total reflection infrared (ATR IR) spectroscopy combined with supervised machine learning for intraoperative liver tumor classification based on a holistic biochemical signature approach. Fresh liver tissue from 69 surgical patients was analyzed using a probe consisting of Ge ATR crystal and silver halide fibers. Supervised algorithms reliably classified normal tissue and tumor subtypes (HCC, CCC, metastases) using cross-validation and independent test sets. Normal liver tissue was distinguished primarily by differences in glycogen content and structural compactness of tumor tissue. Normal and tumor tissues were differentiated with a sensitivity of 0.89 and a specificity of 0.92. The accuracy of spectroscopic classification is 0.90. The three-group classification of tumor subtypes also yielded an average accuracy of 0.90. HCC is characterized by a higher glycogen content compared to CCC and metastases and can be identified spectroscopically with high reliability. CCC showed distinct protein-associated spectral signatures, while metastases exhibited unique profiles reflecting their different origins. In a minority of cases, misclassifications occurred, indicating potential for further refinement. Fiber-based ATR IR spectroscopy in combination with machine learning provides a rapid, objective, and highly accurate intraoperative tool for liver tumor classification. This label-free biochemical approach may enhance surgical precision and reduce recurrence risks across the full range of solid tumor entities.

**Supplementary Information:**

The online version contains supplementary material available at 10.1038/s41598-025-06250-z.

## Introduction

Liver cancer poses a major challenge to global health, with hepatocellular carcinoma (HCC) and cholangiocellular carcinoma (CCC) being the most common primary liver malignancies. These cancers rank among the seven leading causes of cancer-related deaths in both men and women, with an average 5-year survival rate of only 22%^1^. Additionally, metastases from other organs frequently affect the liver. The accurate intraoperative identification and discrimination of these tumor types are critical for effective surgical intervention and improved patient outcomes. Surgical tumor resections of HCC, CCC and liver metastases require a precise delineation of malignancy margins to reduce the risk of recurrent tumor growth while preserving as much healthy tissue as possible. This is particularly crucial regarding the infiltrative nature of liver tumors, which often extend beyond visible borders^2^.

Current state-of-the-art for the intraoperative assessment of tumor margins relies primarily on frozen section analysis. This technique involves rapid tissue freezing, sectioning, and histopathological evaluation. Although effective, limitations of this method are obvious. It depends on staining and labelling; it is time- and staff-consuming and of subjective fashion. However, error rates of frozen section analysis in hepato-pancreato-biliary histopathology compared to permanent, paraffin-embedded analysis are reported to be around 2%. Yet, most mistakes are made due to misinterpretation and lack of pathologist’s experience^3–5^.

Hence, there is a pressing need for real-time, objective, and label-free methods that can assist surgeons in rapid and accurate discrimination between tumor entities during liver cancer surgery inside the operation room.

In recent years, molecular spectroscopy has emerged as a promising tool in cancer diagnostics due to its ability to provide detailed molecular information based on the characteristic molecular vibrations of tissue components^6–9^. In infrared (IR) spectroscopy, IR light interacts with the molecules in biological tissues, producing unique spectral fingerprints that correspond to specific molecular structures, such as proteins, lipids, carbohydrates, and nucleic acids. These fingerprints can be used to differentiate between normal and malignant tissues, as well as between various types of tumors^9–11^. One of the key advantages of IR spectroscopy is its ability to perform these analyses without the need for tissue staining or preparation, making it ideal for intraoperative applications.

Recent advances in fiber-optic technology have enabled the adaptation of IR spectroscopy for real-time tissue analysis in the operating room with the use of flexible optical fibers, leading to direct interaction between tissue classification and surgical team without disrupting surgical workflow.

The incorporation of machine learning algorithms into the IR spectroscopic framework enhances the diagnostic potential of this technique^9,11^. Supervised classification models can process the complex and high-dimensional spectral data to classify different types of cancer tissues accurately. By training the models on known tissue spectra, a classification system can be created, distinguishing between benign and malignant tissue.

In the presented study, fiber-optic IR spectroscopy in combination with supervised classification models was employed to perform label-free intraoperative discrimination of different liver tumor entities. This approach leverages the capability of attenuated total reflection (ATR) IR spectroscopy, a technique of IR spectroscopy utilizing an ATR crystal to acquire information about the selective absorption of IR light of the tissue sample. The ATR technique provides detailed information about both cellular components and the extracellular matrix (ECM) by allowing the penetration of IR radiation up to a few micrometers into the tissue.

The current research demonstrates:

- the feasibility of using fiber-optic IR spectroscopy for the intraoperative discrimination of primary and secondary liver tumors.
- a rapid, non-invasive, objective, label-free and highly accurate method for distinguishing between different tumor types based on their molecular composition.
- the diagnostic capabilities by integrating machine learning algorithms into spectroscopic analysis, paving the way for its implementation into surgical practice.

## Results

### FT-IR spectroscopic imaging of thin sections

The first question is whether normal liver tissue can be distinguished from tumor tissue based on IR spectra. Previous studies on the IR spectroscopy-based identification of tumor tissue have often shown that tumors are heterogeneous. For example, areas of normal tissue may be embedded, or the tumor may be interspersed with newly formed connective tissue. Therefore, in a first step, thin sections of the tissue samples were prepared and analyzed using IR spectroscopic imaging.

**Fig. 1:**
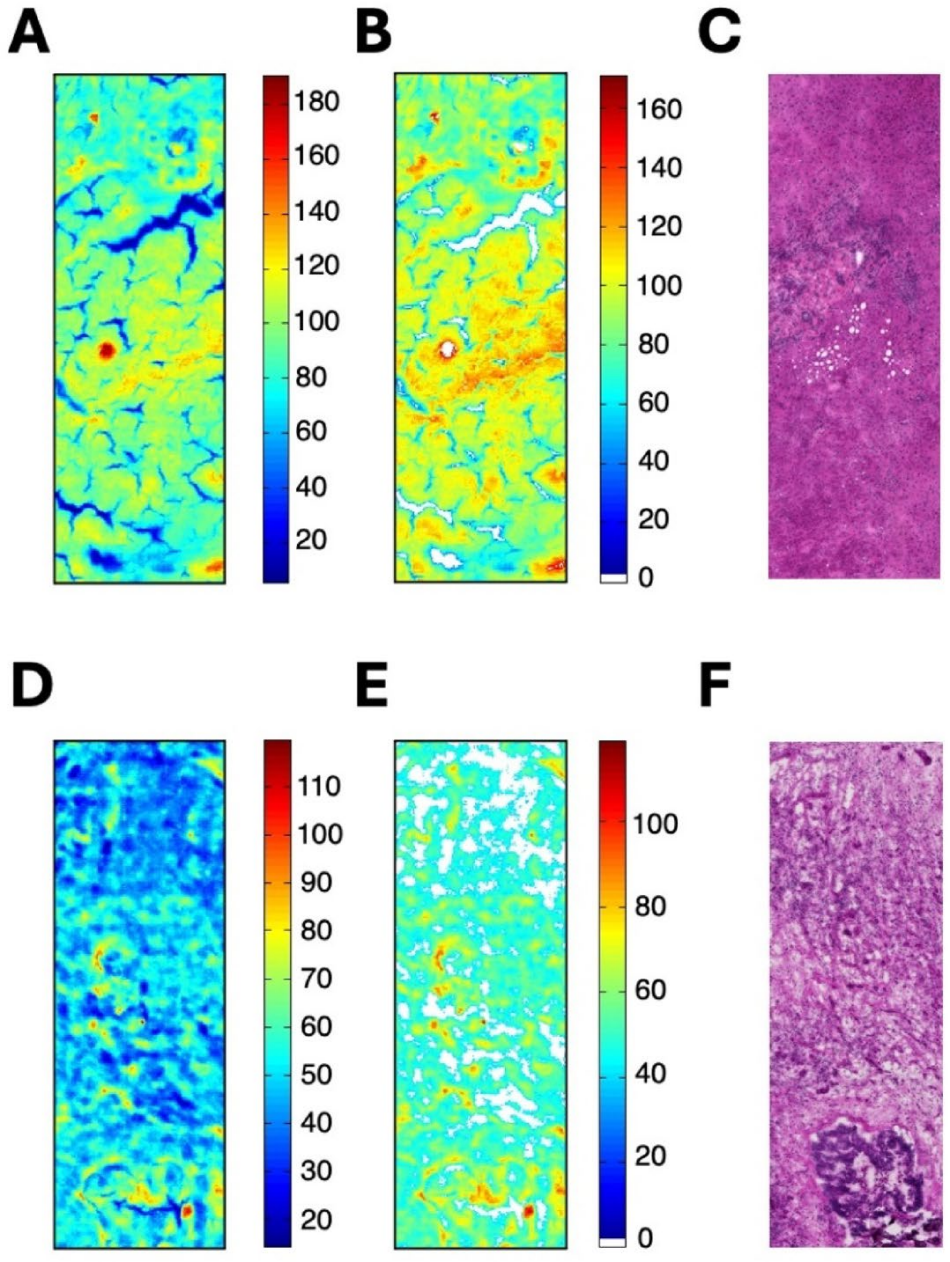
IR spectroscopic and H&E-stained images of normal liver tissue (top row) and tumor tissue (bottom row, metastasis of rectal cancer). The contrast in all spectroscopic images is based on the sum of the absorbance values in the spectral region from 950 cm^− 1^ to 1800 cm^− 1^. The dimension of the images is 700 × 2100 μm. (**A** and **D**): spectroscopic images of the raw spectra, (**B** and **E**): spectroscopic images after selection of the spectra, (**C** and**F**): corresponding H&E-stained images.

Figure 1 shows a representative example of normal liver tissue and tumor tissue (metastasis of rectal cancer) of the same patient. The spectroscopic images calculated from the raw spectra are shown in Fig. 1A and D, respectively. Spectra that did not meet the criteria (see Materials and Methods) were removed from the data set. The spectroscopic images, shown in Fig. 1B and E, were calculated with the pre-processed data. White pixels indicate outlier spectra that were removed from the data set. Figure 1C and F show the Hematoxylin and eosin (H&E) images of the consecutive tissue sections. The tumor tissue (see Fig. 1F) is apparently not characterized by a particularly large heterogeneity such as a gland formation. The same can be concluded for normal tissue sample. It can therefore be assumed that point measurements also provide representative information regarding the entity.

**Fig. 2:**
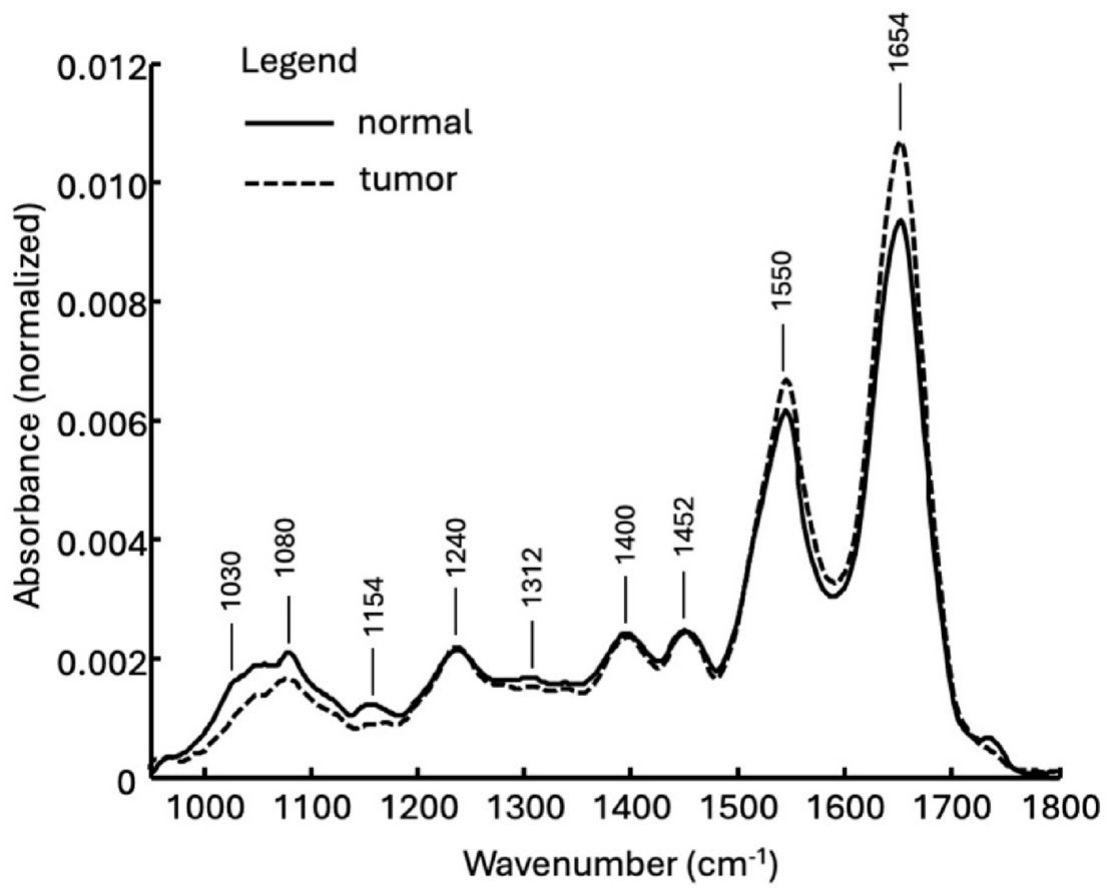
Mean spectra, calculated from the area-normalized spectra of normal and tumor tissue.

The differences between normal and tumor tissue can be observed easily using the mean spectra. In Fig. 2, the calculated mean spectra from the corresponding data sets of Fig. 1B and E are plotted. In order to compensate for variations in the thickness of the cryo sections, all spectra were first area-normalized and then the respective mean spectra were calculated. It should be noted that due to the biochemical complexity of the tissue samples, overlapping spectral features cannot be separated or deconvoluted. The spectra of both entities are dominated by the amide-I and amide-II bands at 1654 cm^− 1^ and 1550 cm^− 1^ respectively. The bands at 1400 cm^− 1^ and 1452 cm^− 1^ can be assigned to vibrations of the CH_x_ groups. The weak signal at 1312 cm^− 1^ and the slightly stronger band at 1240 cm^− 1^ are assigned to the amide-III vibration mode. The band at 1154 cm^− 1^ is assigned to the antisymmetric stretching vibration mode of the CO-O-C group of glycogen^12^. The shoulder at about 1030 cm^− 1^ can also be assigned to glycogen and originates from the C-O stretching vibration^12,13^. At 1080 cm^− 1^, the vibration of the phosphate groups of the DNA and phospholipids is particularly prominent.

The mean spectra show that liver tumor has a lower glycogen content than normal tissue. In addition, the level of proteins is higher in tumor tissue compared to normal tissue. The lower glycogen content and the variations in the protein profile alone should lead to the identification of tumor tissue when spectra of fresh tissue samples are analyzed.

## ATR fiber-based spectroscopy of native surgical resection specimens

**Fig. 3:**
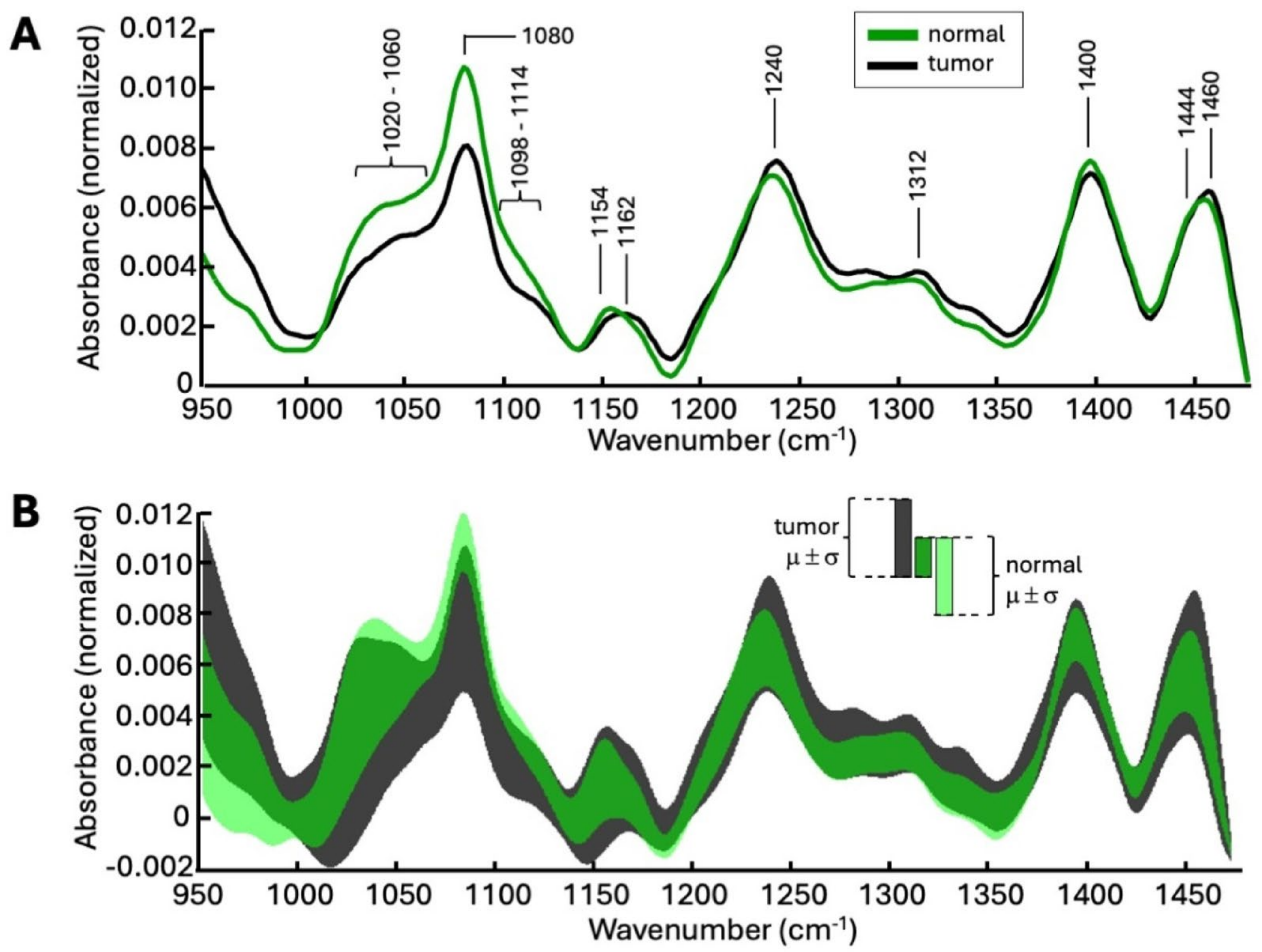
**(A)** Calculated mean IR ATR spectra of native normal tissue (green) and of tumor samples (black). **(B)** Plot of the two-sided standard deviation (±σ) to the mean value (µ). Normal tissue: light green and tumor tissue: dark gray. The overlay of both µ ±σ bands is shown in dark green.

Figure 3A shows the calculated mean ATR spectra of normal tissue samples and pathological tissue, i.e. all tumor samples. At first glance, the spectral profiles of both mean spectra are similar. This is particularly true for bands at 1460 cm^− 1^ and 1400 cm^− 1^. Both absorption bands can be assigned to the antisymmetric or symmetric deformation modes of the CH_3_ groups. The comparatively weak band at 1312 cm^− 1^ as well as the stronger band at 1240 cm^− 1^ are assigned to the amide III vibration mode. However, it should also be mentioned that the band at 1240 cm^− 1^ also represents components of the phosphate vibrations of nucleic acids. The weak band at 1162 cm^− 1^ represents vibrations of the C-OH groups mainly arising from amino acids. It should be noted that this comparatively broad band is composed of several components. In addition to the vibrational modes of different amino acids, polysaccharides also contribute to this signal. The stronger signal of normal tissue samples at 1154 cm^− 1^ is most likely associated with glycogen. This is also supported by the absorption bands between 1098 cm^− 1^ and 1114 cm^− 1^, and especially in the range from 1020 cm^− 1^ to 1060 cm^− 1^. In this range, particularly strong and broad absorption bands of glycogen occur. The band at 1080 cm^− 1^ represents vibrations of phosphate groups of DNA but also of phospholipids. Normal tissue shows a larger signal here than tumor tissue. The higher absorption of normal tissue at 1080 cm^− 1^ is due to a more compact and dense cell structure since the genome of normal cells and tumor cells is identical. Liver tumor tissue is generally less compact and more loosely structured. The resulting lower cell density leads to a weaker signal from phosphate groups of DNA and phospholipids. In general, the plots show that normal liver tissue samples have a higher glycogen content than tumor samples. Figure 3B shows the band of the sample standard deviation (positive and negative) for both tissue classes and their overlap. Except for a few spectral ranges, the scattering of the spectra is so strong that even the simple standard deviations overlap, making an easy separation of the tissue spectra, for example using separate bands, impossible. Instead, multivariate analysis and supervised classification methods must be used because of the strong scattering of absorbance values.

**Fig. 4:**
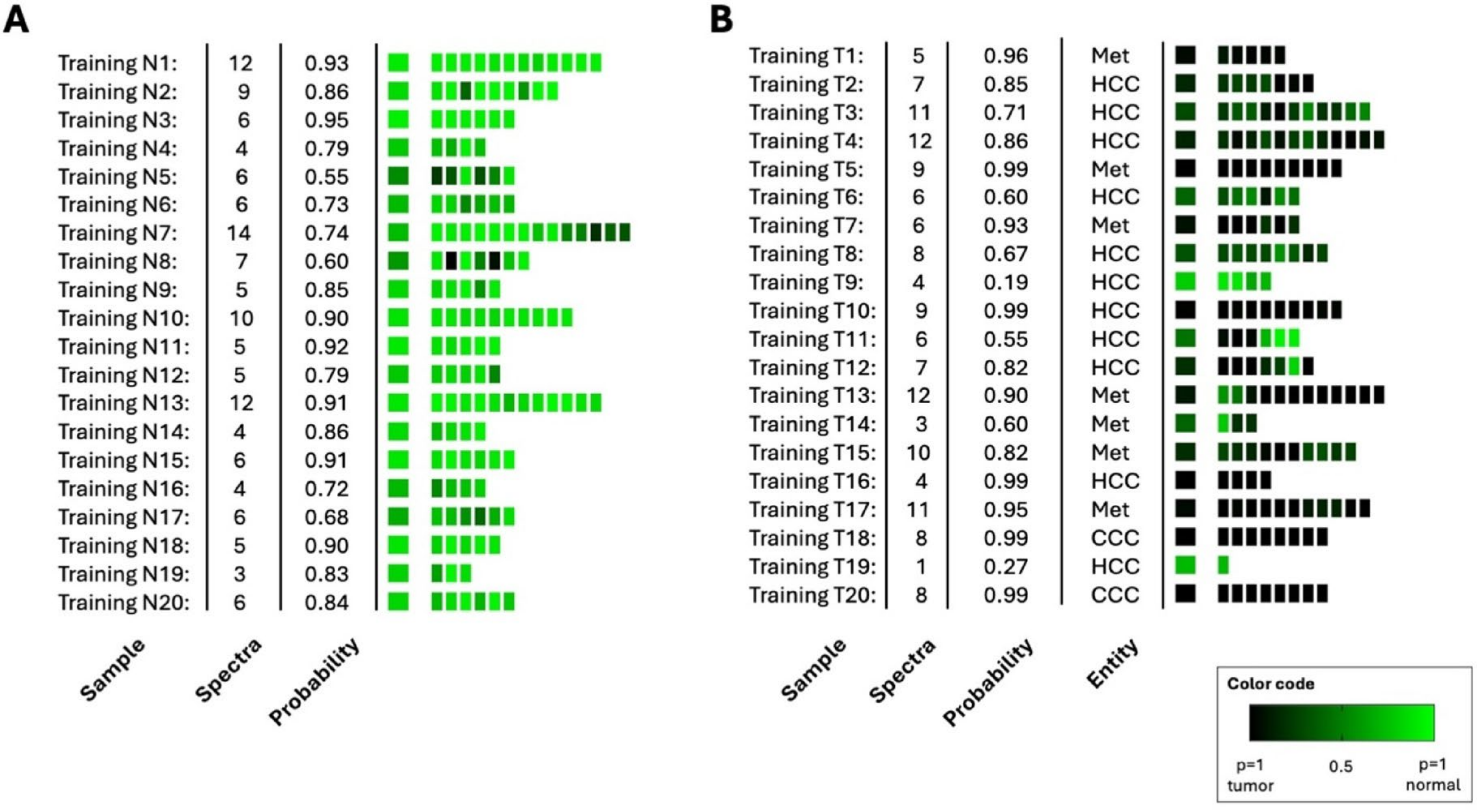
Supervised re-classification result of the training set **(A)** for normal tissue samples and **(B)** for tumor samples. The three pathologies metastases, CCC and HCC were combined into one tumor class. The left column indicates the patient number, followed by the number of collected spectra and the calculated mean probabilities of class. Mean probability of class assignment calculated from the individual classified spectra is shown in the large colored boxes on the left. Classified individual spectra are represented by the smaller fields on the right. The probability of class membership is indicated by the color code black - green.

**Fig. 5:**
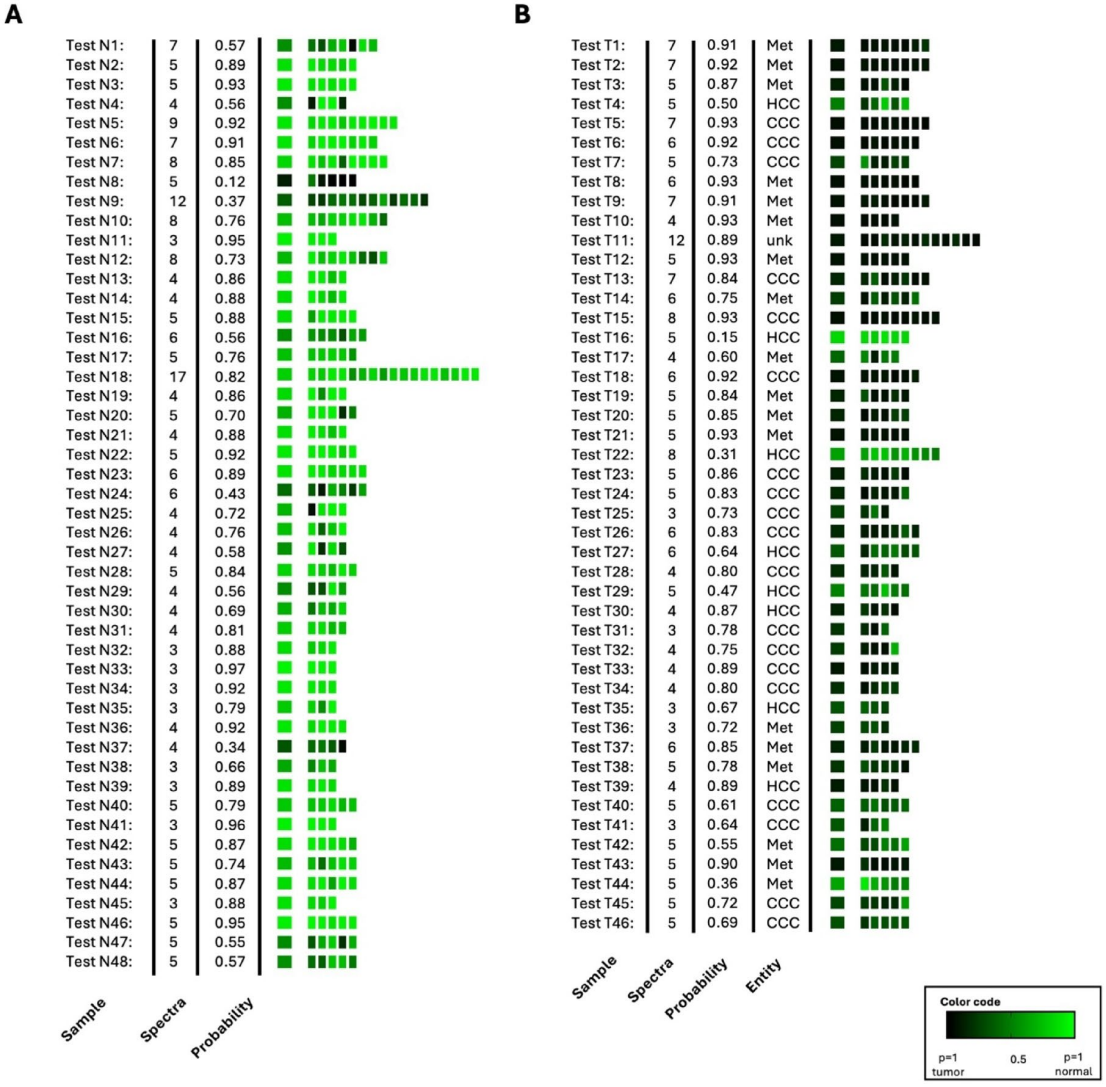
Supervised classification result of the independent test set **(A)** for normal tissue samples and **(B)** for tumor samples. The left column indicates the patient number, followed by the number of collected spectra and the calculated mean probabilities of class. Mean probability of class assignment calculated from the individual classified spectra is shown in the large colored boxes on the left. Classified individual spectra are represented by the smaller fields on the right. The probability of class membership is indicated by the color code black – green. Met – Metastasis, unk – unknown.

Figure 4 A and 4B show the re-classification of the training set. A tissue sample was classified as correct for the respective class if the calculated mean probability was *p* > 0.5. All normal tissue samples were correctly classified. Two of the tumor samples were misclassified. The supervised classification is based on three general spectral ranges. The algorithm used the ranges between (i) 995 cm^− 1^ − 1005 cm^− 1^, (ii) 1102 cm^− 1^ − 1114 cm^− 1^ and (iii) 1151 cm^− 1^ − 1155 cm^− 1^. The first range between 995 cm^− 1^ − 1005 cm^− 1^ cannot be assigned to a specific vibration mode but indicates obviously a different water content of the samples. The other two ranges are associated with glycogen and underline the hypothesis that the differentiation between normal and tumor tissue is mainly due to the different glycogen content. Similar to the re-classification of the training set (see Fig. 4) the classification of the independent test set is shown in Fig. 5. Four normal tissue samples were assigned to the tumor class and 5 tumor sample to the normal class. The confusion matrix for the test set is shown in Table 1. The classification results show a sensitivity of 0.89 and a specificity of 0.92. This results in an accuracy of 0.90 and a calculated F1 score of 0.90.

**Table 1:** Confusion matrix for the classification of the test set of normal and tumor tissue.

	Normal tissue	Tumor tissue
classified as normal tissue	44	5
classified as tumor tissue	4	41

The misclassified samples of normal tissue can be explained by the training set. HCC tumors and normal tissue show a greater absorbance in the range of 1020 cm^− 1^ to 1060 cm^− 1^ and at 1080 cm^− 1^ compared to CCC tumors and metastases. If spectra of normal tissue in the test set are more similar to those of HCC tumors, the spectra are classified as tumor. Due to the high similarity of the spectral profiles of normal tissue and HCC tumors, a dedicated separation of only these two classes may be advisable. Furthermore, recent studies in the era of multiomic diagnostic revealed several subtypes of HCC, partly not distinguishable within histopathological stainings^14,15^. However, this must be investigated in a separate larger study. To determine whether the spectral regions identified by the classification algorithm are significantly different, all spectra of the test sets were tested using a two-sample t-test. The results of the t-test for each data point of the spectra of normal tissue compared to the spectra of tumor tissue are shown in Supplementary Fig. 1.

The question that now arises is whether not only normal tissue and tumor can be distinguished, but also whether a differentiation within the tumor class is possible, i.e. into metastases, CCC and HCC tumors.

**Fig. 6:**
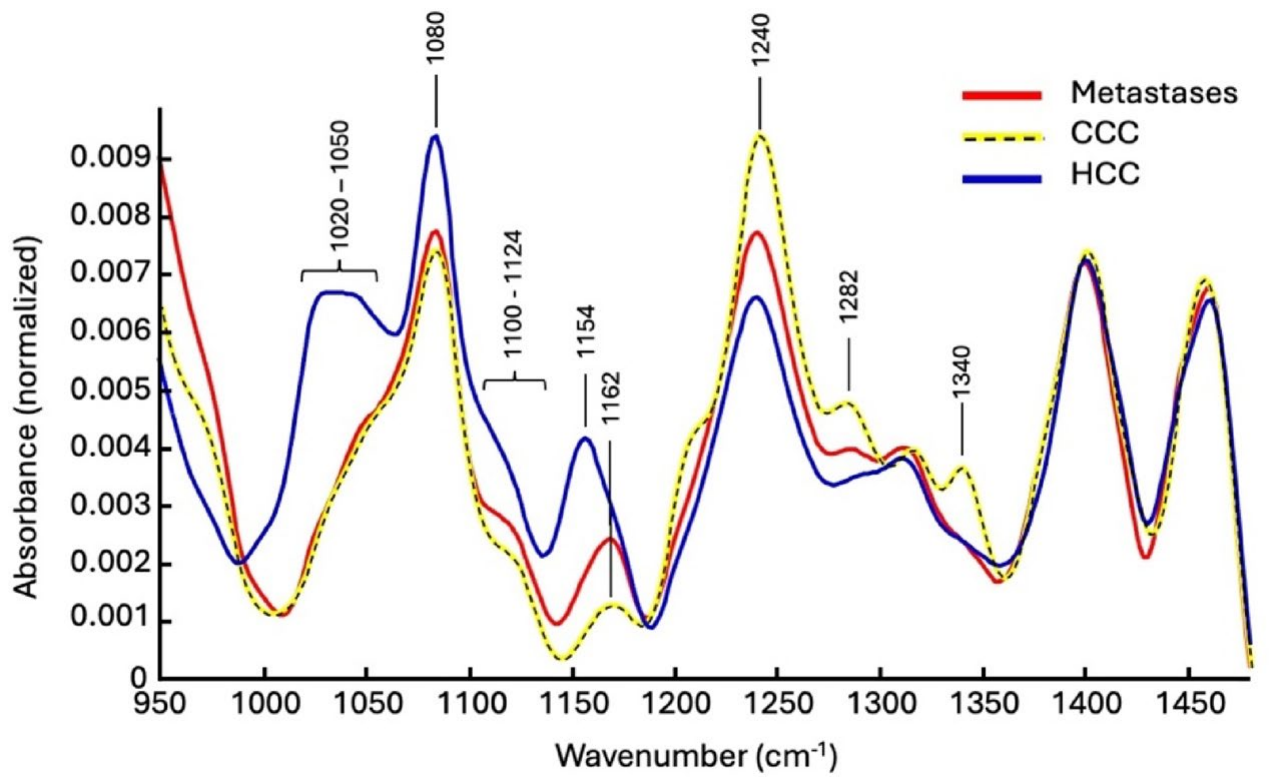
Calculated mean value spectra for the three pathological classes. For better visibility, the yellow mean value spectrum of the CCC class was underlaid with a black dashed line.

Figure 6 shows the mean spectra of the three classes calculated from the individual measured spectra. At first glance, there are clear differences, particularly in the spectral ranges representing glycogen. The differences in the range from 1150 cm^− 1^ to 1190 cm^− 1^ are very noticeable. HCC tumors therefore have a higher average glycogen level than metastases and CCC tumors. Higher levels of glycogen in HCC tumors compared to metastases or CCC tumors can be explained considering the different nature of tumors. HCC tumors arise from hepatocytes which accumulate glycogen; therefore, this is observable from the spectra. The average content of glycogen is similar for metastases and CCC tumors. Furthermore, differences are also recognizable for the amide-III vibration mode centered at 1240 cm^− 1^. It should be mentioned that the broad signal with a center at 1240 cm^− 1^ is composed of different components. These include mainly collagen, the amide III vibration mode of other proteins and the antisymmetric stretching vibration of phosphate groups, as well as a vibration mode of phosphodiesters^16^. The higher signal of CCC is probably due to an altered protein profile. The biochemical nature of this is not yet fully understood. Additionally, the bands at 1282 cm^− 1^ and 1340 cm^− 1^ are collagen-associated. Increased collagen levels are related to growth of the desmoplastic stroma of the tumor which is rich in connective tissue containing collagen. This desmoplastic reaction is a common feature of CCC tumors^17–20^. Finally, all three classes show very similar mean absorbance profiles in the range from about 1360 cm^− 1^ to 1480 cm^− 1^, where mainly CH_2_ and CH_3_ groups show absorptions.

To further analyze the three classes and ultimately examining the possibilities of separating them based on their spectral profile, bands of the symmetric standard deviation were plotted (Supplementary Fig. 2). The graphical representation of the superimposed standard deviations is displayed. Obviously, HCC tumors can be identified by their higher glycogen content in the range 1000 cm^− 1^ to 1050 cm^− 1^, while CCC tumors can be identified in particular by the intensity of amide-III vibration mode at 1240 cm^− 1^ and the collagen bands at 1282 cm^− 1^ and 1340 cm^− 1^. However, a comparatively strong scattering of the absorption values can also be seen here, so that a supervised classification routine has to be used.

**Fig. 7:**
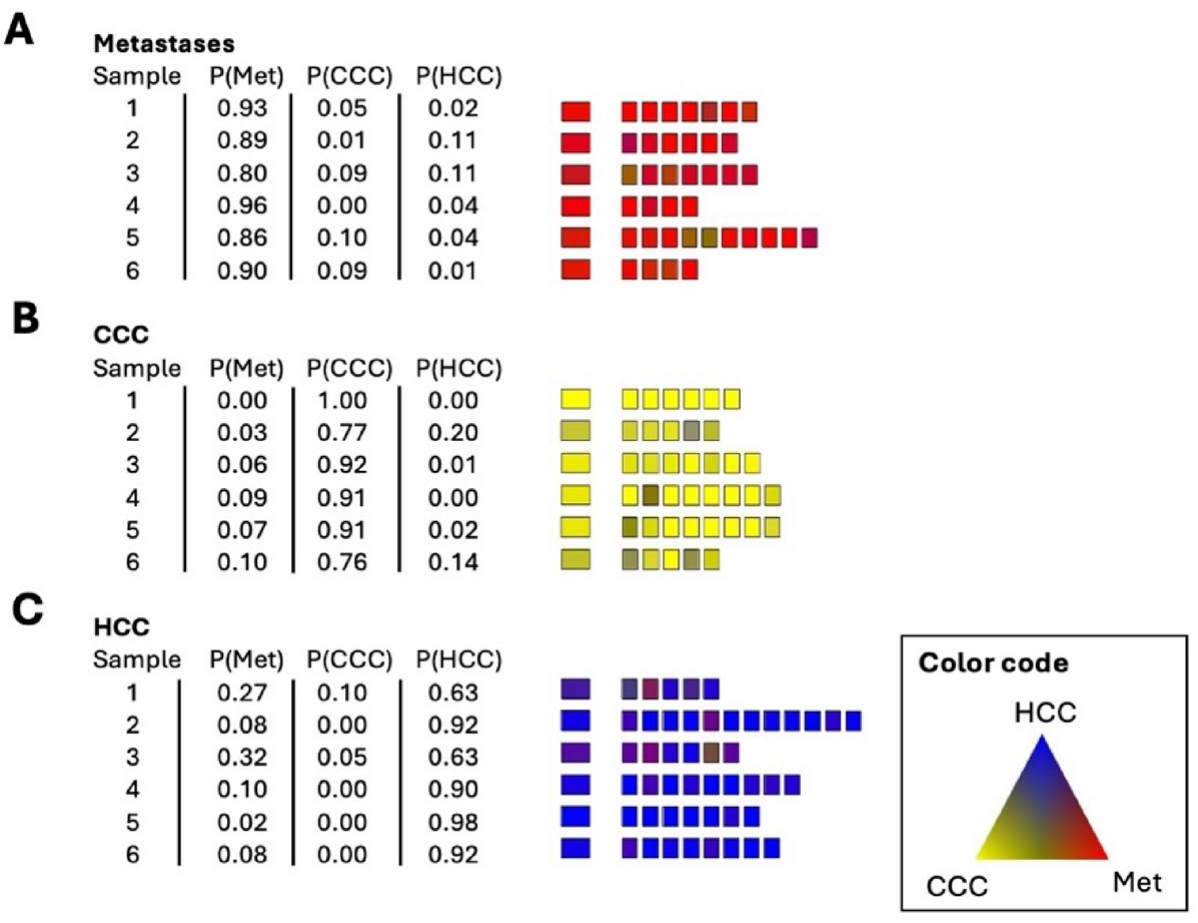
Supervised re-classification result of the training set **(A)** metastasis (Met), **(B)** CCC tumor and **(C)** HCC tumor. The left column indicates the patient number, followed by the calculated mean probability values (P) for the three classes. Mean probability of class assignment calculated from the individual classified spectra is shown in the left-sided large box column. Classified individual spectra are represented by the smaller fields on the right. The probability of class membership is indicated by the red-yellow-blue color code. Met – Metastasis.

Corresponding to Fig. 4A and B, the results of the training set are shown in Fig. 7. The highest calculated probability for a class defines the class membership. None of the calculated mean values of the probability of class membership shows a misclassification. The spectral regions selected by the algorithm are 968 cm^− 1^ − 972 cm^− 1^, 1252 cm^− 1^ − 1258 cm^− 1^ as well as 1396 cm^− 1^ and 1450 cm^− 1^. The range between 968 cm^− 1^ and 972 cm^− 1^ cannot be assigned to any specific vibration mode. Presumably there is a different water content indicated. The range from 1252 cm^− 1^ to 1258 cm^− 1^ is attributed to glycogen. The frequencies of 1396 cm^− 1^ and 1450 cm^− 1^ are associated with deformation modes of CH_3_ groups and point to variations of the protein profile.

**Fig. 8:**
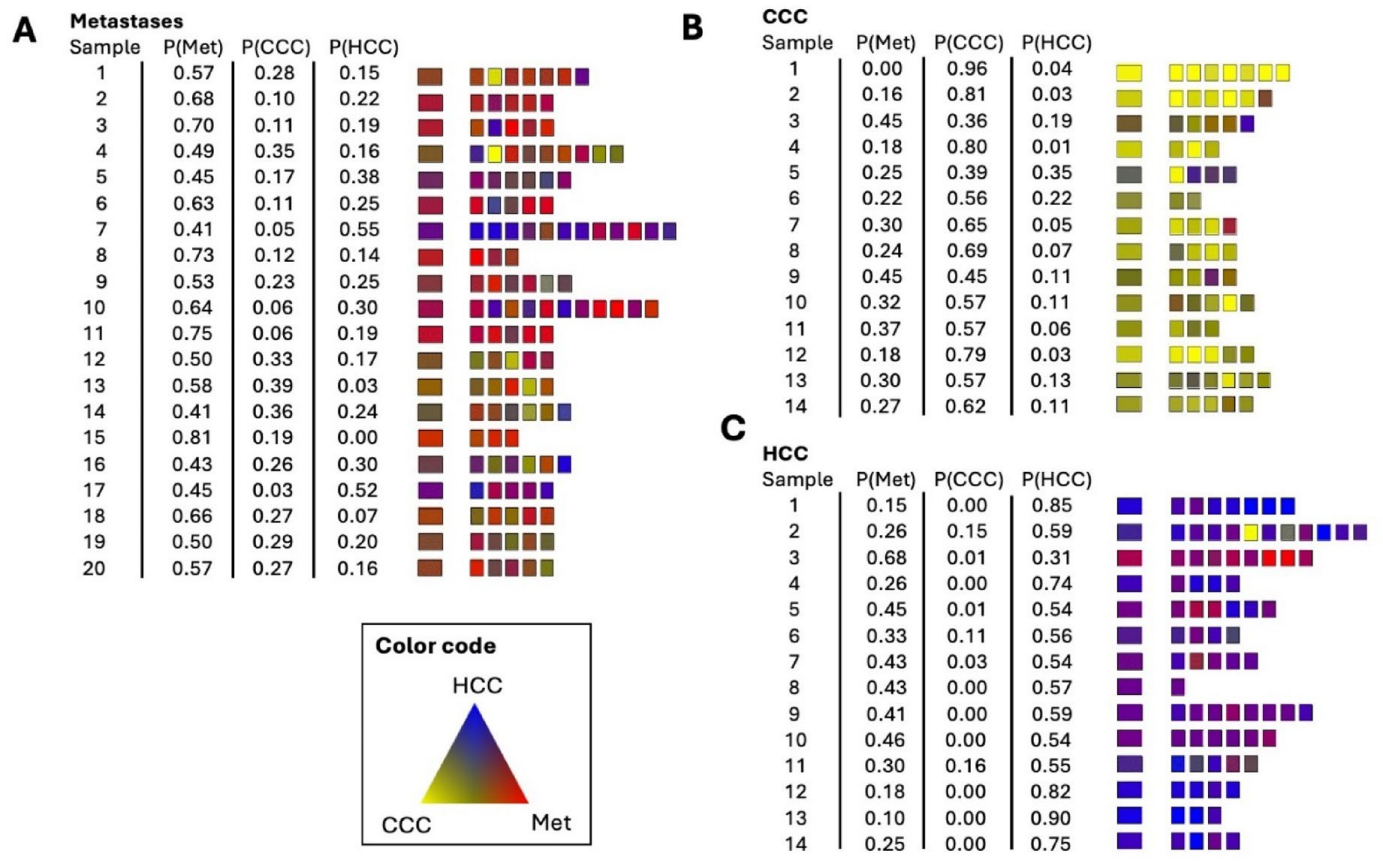
Supervised classification result of the independent test **(A)** metastasis (Met), **(B)** CCC tumor and **(C)** HCC tumor. The left column indicates the patient number, followed by the calculated mean probability values (P) for the three classes. Mean probability of class assignment calculated from the individual classified spectra is shown in the left-sided large box column. Classified individual spectra are represented by the smaller fields on the right. The probability of class membership is indicated by the red-yellow-blue color code. Met – Metastasis.

The classification of the independent test set is shown in Fig. 8. The highest of the three calculated mean probability values P(Met), P(CCC), and P(HCC) determines the assignment to the class. Table 2 shows the confusion matrix for the 3-group classification of the test set of CCC, HCC and metastases.

**Table 2:** Confusion matrix for the classification of the test set of CCC, HCC, and metastases.

	CCC	HCC	Metastases
Classified as CCC	13	0	0
Classified as HCC	0	13	2
Classified as metastasis	1	1	18

A detailed investigation of the causes of the misclassification is required, but this is only possible with a larger number of samples.

The spectra of the test set were also tested to see whether they differed between CCC, HCC and metastases. Therefore, three paired t-tests were performed. The results are shown in Supplementary Fig. 3.

## Discussion

The current study demonstrates a novel application of fiber-based ATR IR spectroscopy combined with machine learning approaches for the intraoperative discrimination of liver tumor entities. This strategy addresses the critical need for real-time, label-free, and objective methods for tumor classification during surgical interventions. Compared to traditional histopathological techniques, such as frozen section analysis, this spectroscopic method significantly reduces the dependency on time-consuming staining processes and subjective interpretation, thereby optimizing the surgical workflow and improving precision in tumor resection. Recent pilot studies showed better results in ATR sample analysis compared to frozen section analysis^11^. This ATR IR spectroscopic method represents an important step towards the advancement in intraoperative diagnostic capacity due to its capability of differentiating primary liver tumors, such as HCC and CCC, as well as metastatic liver lesions, based on biochemical composition. Recently, hyperspectral imaging in combination with a convolutional neuronal network was reported to be able to differentiate between cirrhotic liver specimens and HCC tumors with an accuracy of up to 90%. However, these results were gained retrospectively from specimen slides in a laboratory workflow^21^.

In contrast to conventional imaging or histological methods, fiber-based ATR IR spectroscopy provides direct insights into molecular properties without focusing on spatial distribution of cellular components or phenotype features.

The combination of supervised classification models with spectroscopic frameworks enhances its clinical potential allowing not only the distinction between tumor and non-tumor lesions but also to discriminate different liver tumor entities – even in the presence of high rates of biovariability.

The study provided the following insights:

i) The IR spectroscopic differentiation between normal liver tissue and tumor tissue is mainly based on the different glycogen content and the different tissue structure. In average, normal tissue has a higher glycogen level, especially compared to CCC and metastases. The loose and less dense tissue structure of tumors leads to weaker absorption signals from phosphate groups. Both biochemical and spectral characteristics allow a reliable distinction between normal and tumor tissue. Especially the differentiation of HCC from normal liver tissue shows some obstacles for the algorithm. However, histopathological evaluation of small liver samples may also challenge pathology^22^.
ii) HCC can be distinguished from CCC and metastases mainly by the higher glycogen content. In general, the biochemical profile of HCC is similar to that of normal tissue.
iii) Metastases show a different protein profile than CCC and HCC tumors. This is particularly evident from the spectral bands at 1162 cm^− 1^ and 1240 cm^− 1^.
iv) CCC show strong signals at 1240 cm^− 1^, setting them apart from all other entities. This indicates a higher content of proteins but also a possible increased occurrence of phosphodiesters.

It should be noted that with only 6 patients each, the training set is relatively small. Nevertheless, the classification of the test set with 5 misclassifications of a total of 46 samples is promising. In order to make the method more reliable and robust and ultimately transfer it to practical application, the size of the training set must be increased.

The implications of this technique extend beyond liver surgery. By offering rapid and robust feedback with a probability score for entity membership, this approach may guide surgeons to achieve more precise tumor resection margins, thereby reducing the risk of recurrence and persevering physiological and healthy tissue. Recent studies have already shown that this methodology can be adapted for other types of cancer requiring precise tumor delineation, including pancreatic and kidney tumors^10,11^. However, the current study is the first to show the capability to differentiate even several tumor classes within one organ. Given the potential of further delineation of subgroups within one single tumor entity, even intraoperative therapy planning including immunotherapy and chemotherapy for personalized treatment becomes feasible.

Furthermore, integrating fiber-optic probes into existing surgical workflows for the application of molecular spectroscopic analysis inside the human body during surgery seems reachable. In such implementation, this tool has the potential to become a standard adjunct in oncological surgeries, enhancing both diagnostic accuracy and surgical precision. Unlike methods that rely on near-infrared cameras, preoperative MRI or fluorescence markers, the ATR IR spectroscopy method combines a spatial resolution of a few millimeters with native, unstained tissue analysis on a molecular level, without the need for specialized cameras or similar imaging equipment^23–26^. In general, the study shows that tissue classification on freshly resected tissue using IR fiber spectroscopy is possible. For in situ applications, further approvals (e.g. certified sterilizability of the fiber probe) need to be obtained.

Considering the heterogeneity of the tissue samples, all specimens were cut several times and spectra were obtained from different parts of the sample resulting in multiple spectra from one sample. The final class assignment was determined by the highest value of mean probabilities.

While the results are promising, several limitations must be addressed before widespread clinical adoption. Notably, the strong overlap in some spectral ranges and the need for multivariate analysis highlight the complexity of interpreting IR spectral data. Although machine learning enhances accuracy, the models rely heavily on the quality and diversity of the training datasets. Expanding the dataset to include more patients and tumor subtypes will be essential for improving robustness and generalizability. Additionally, factors such as tissue hydration, temperature, and pressure applied to the sample during the measurements may introduce variability. To reduce the impact of these factors, the measurements were performed according to a standard protocol provided in the supplementary material section. Standardizing these parameters will be crucial for consistent performance. Moreover, while the study demonstrated high accuracy, occasional misclassifications were observed in independent test sets, suggesting that further refinement of algorithms and optimization of spectral ranges is required.

## Conclusion

Fiber-based ATR IR spectroscopy combined with machine learning models represents a significant innovation in intraoperative cancer diagnostics. Its ability to provide rapid, objective, and molecularly detailed tissue classification enhances its clinical utility, particularly in liver surgery, but also in other solid cancer entities. By addressing current limitations and expanding clinical validation, this method holds significant promise for revolutionizing surgical oncology.

## Methods

### Sample collection

Liver tissue samples were collected from surgical liver resections at the Department of Visceral, Thoracic and Vascular Surgery of the University Hospital Carl Gustav Carus, Technische Universität Dresden. Informed consent was obtained from all patients prior to surgery. Surgical procedures were performed for medically indicated reasons. No additional tissue was resected for research purposes. Specimens were examined by a board-certified pathologist. A total number of 69 patient samples were examined for spectroscopic analysis.

## H&E staining

For internal validation of class membership, cryo-sectioned tissue (10 μm) was stained by H&E for gold standard histopathological analysis. The defrosted and dried frozen sections were treated with 70% ethanol for 30 s. After a short washing step with distilled water, hematoxylin was applied for 3.5 min, followed by rinsing in distilled water and hydrochloric acid solution. The tissue was blued in warm water for 5 min and subsequently treated with eosin for 15 s. Before embedding the stained section in Entellan™ new (1.07961, Sigma-Aldrich, Saint Louis, USA), rinsing and dehydration with 70% and 90% ethanol, 2-propanol and xylene were performed.

## FT-IR spectroscopic imaging

FT-IR spectroscopic images were collected in transmission mode using a FT-IR spectrometer Vertex 70 coupled with an infrared microscope Hyperion 3000 (Bruker Optik GmbH, Ettlingen, Germany) and a MCT focal plane array detector. The 15-fold Cassegrainian objective with a numerical aperture of 0.4 imaged a sample area of approx. 175 × 175 µm^2^. The imaging detector was a Santa Barbara Focalplane (Goleta, CA, USA) MCT 64 × 64 array detector. From each sample 4 × 12 individual FT-IR spectroscopic images with a total size of 700 × 2100 µm^2^ were captured. Reference spectroscopic images were recorded from the pure calcium fluoride window. For all measurements, a total number of 100 interferograms (scans) were combined. The interferograms were Fourier transformed applying Happ-Genzel apodization and zero filling factor of 1. The resolution of the raw spectra was 6 cm^-1^. Spectra of the sample spectroscopic image were ratioed against the spectra of the reference image and converted into absorbance values. As the only step in the data preprocessing, outliers were removed from the data sets and replaced with white pixels in the evaluated spectroscopic images. Spectra with a sum of all absorbance values of less than 20 or whose absorbance value of the amide I band at 1650 cm^-1^ was less than 0.01 were declared as outlier and removed from the data set. These spectra do not contain any relevant information and are mostly attributable to gaps in the tissue section.

### Fiber-based IR ATR spectroscopy

Measurements of freshly resected human liver tissue spectra were performed by using a fiber-based spectroscopy probe consisting of a Ge ATR crystal and silver halide fibers (Art Photonics GmbH, Berlin, Germany) attached to a FT-IR spectrometer Alpha (Bruker Optik GmbH, Ettlingen, Germany), equipped with an external liquid nitrogen cooled mercury–cadmium–telluride detector (Model IRA-20-00131, Infrared Associates, Inc.). The procedure of tissue spectra measurements in detail is described elsewhere^11^. Briefly, samples received from the surgical team were sliced into several pieces and measurements were performed from the freshly cut surface of each slice within the operating room tract. The diameter of analyzed sample surface area was 900 μm^11^. Number of slices and analyzed spectra varied depending on the size of the sample, usually from 3 to 8 cuts per tissue sample. The fiber probe was smoothly pressed against the freshly cut surface area for recording spectra. The recording time for each spectrum was approximately 1 min. These spectra were labelled as individual spectra further in this manuscript.

Fifty interferograms were recorded and Fourier transformed to single beam spectrum using Blackmann-Haris 3-term apodization function, zero filling factor of 2. The resolution of spectra was 4 cm^− 1^. After Fourier transformation, the single beam sample spectra were ratioed against a background spectrum obtained from the pure Ge ATR crystal and converted into absorbance values. In addition to automatic correction of water vapor bands (atmospheric compensation) preprocessing of the spectra included reducing the spectral range to 950 cm^− 1^ to 1800 cm^− 1^ and a linear two-point baseline correction in this spectral range. The region from 950 cm^− 1^ to 1480 cm^− 1^ was used for classification and the spectra were area normalized to this range.

A detailed protocol for the measurements is added in the supplementary material.

### Data evaluation and classification of ATR spectra

Only the spectral range from 950 cm^− 1^ to 1480 cm^− 1^ was used for further evaluation. In this range there are several bands of biomolecules and only weak absorption bands of water.

The classification for the differentiation of normal tissue and tumor tissue as well as the differentiation of different tumor types were carried out using a supervised algorithm that has been used and described previously^9,11^. The classification is a combination of two algorithms. In a first step, the training data set is divided, with 65% of the spectra being used to determine the classifiers and 35% for subsequent cross-validation. The first algorithm is a genetic routine for selecting the optimal spectral regions^27^. This program uses both the spectra of interest and their gold standard designations (i.e., the histology) as input. Based on this information, the algorithm identifies a series of spectral subregions that together serve as the basis for grouping the spectra according to their histopathological classification. Each spectrum is re-expressed as a series of absorbance values. The classifications were then performed using the quadratic discriminant analysis function *classify* of the Matlab package. The classification result of the training set is verified by k-fold cross validation^28^.

For the classification of normal tissue and tumor tissue, the training set was formed from the spectra of 20 randomly selected patient tissue samples. The normal tissue class comprises 135 spectra and the tumor tissue class 147 spectra representing all three tumor types (HCC, CCC and metastasis). All remaining samples were classified as an independent test set. For the classification of tumor types, a training set consisting of the spectra of 6 randomly selected patients was created. The training set consists of 38 spectra of metastases, 39 spectra of CCC tumors and 37 spectra of HCC tumors. Similar to the discrimination of normal tissue and tumor tissue, all remaining tumor spectra were classified as an independent test set. The *classify* function of Matlab package provides a value that quantifies with which probability the spectrum can be assigned to one of the classes. For graphical illustration, this value was transferred into a color value, whereby the colors are consistently assigned as follows: normal tissue: green, all tumor samples together: black, metastases: red, CCC tumors: yellow and HCC tumors: blue.

All spectra of the training and test sets were min-max normalized, where the minimum is set to zero and the maximum of the absorbance values is equal to one.

Two-sample t-tests were performed using the Matlab function *ttest2*. For each data point (wavenumber), it was tested whether the corresponding absorbances of the area normalized spectra originated from distributions with the same mean value. The significance level was set to 0.05.

## Electronic supplementary material

Below is the link to the electronic supplementary material.

Supplementary Material 1

## Data Availability

Biological data and analytical codes are available upon reasonable request to the corresponding author.
