# Supplementary material for: Algorithm-based intraoperative diagnosis of liver tumors using infrared spectroscopy
Source: Sci Rep. 2025 Jun 20;15:20197. doi: 10.1038/s41598-025-06250-z (PMC12181317; doi:10.1038/s41598-025-06250-z)
Supplement: Supplementary file 1 — Supplementary Material 1 [file 41598_2025_6250_MOESM1_ESM.docx]

***Algorithm-based intraoperative diagnosis of liver tumors using infrared spectroscopy***

Rimante Bandzeviciute^1, 2^; Grit Preusse^2^; Sascha Brückmann^3^; Alexander Hirle^4,5^; Anne Wedemann^4,5^; Franziska Baenke^4,5^; Marius Distler^4,5^; Carina Riediger^4,5^; Jürgen Weitz^4,5^; Valdas Sablinskas^1^; Justinas Ceponkus^1^; Gerald Steiner^2,#^ and Christian Teske^4,5,#,*^

^1^ Institute of Chemical Physics, Faculty of Physics, Vilnius University, Vilnius, Lithuania

^2^ Department of Anesthesia and Intensive Care, Clinical Sensoring and Monitoring, University Hospital and Faculty of Medicine Carl Gustav Carus, Technische Universität Dresden, Germany

^3^ Institute of Pathology, University Hospital Carl Gustav Carus, Dresden, Germany

^4^ Department of Visceral, Thoracic and Vascular Surgery, University Hospital Carl Gustav Carus, Technische Universität Dresden, Germany

^5^ National Center for Tumor Diseases (NCT/UCC), Dresden, Germany: German Cancer Research Center (DKFZ), Heidelberg, Germany; Faculty of Medicine and University Hospital Carl Gustav Carus, Technische Universität Dresden, Dresden, Germany; Helmholtz-Zentrum Dresden - Rossendorf (HZDR), Dresden, Germany

^#^ These authors contributed equally and share senior authorship.

**Supplementary Material**

*** Corresponding author:**

Christian Teske, MD
Department of Visceral, Thoracic and Vascular Surgery

University Hospital Carl Gustav Carus
Technische Universität Dresden
Fetscherstraße 74
01307 Dresden
Germany
Tel: +49 351 458 11909
Email: christian.teske@ukdd.de

**Supplementary Figures**

| 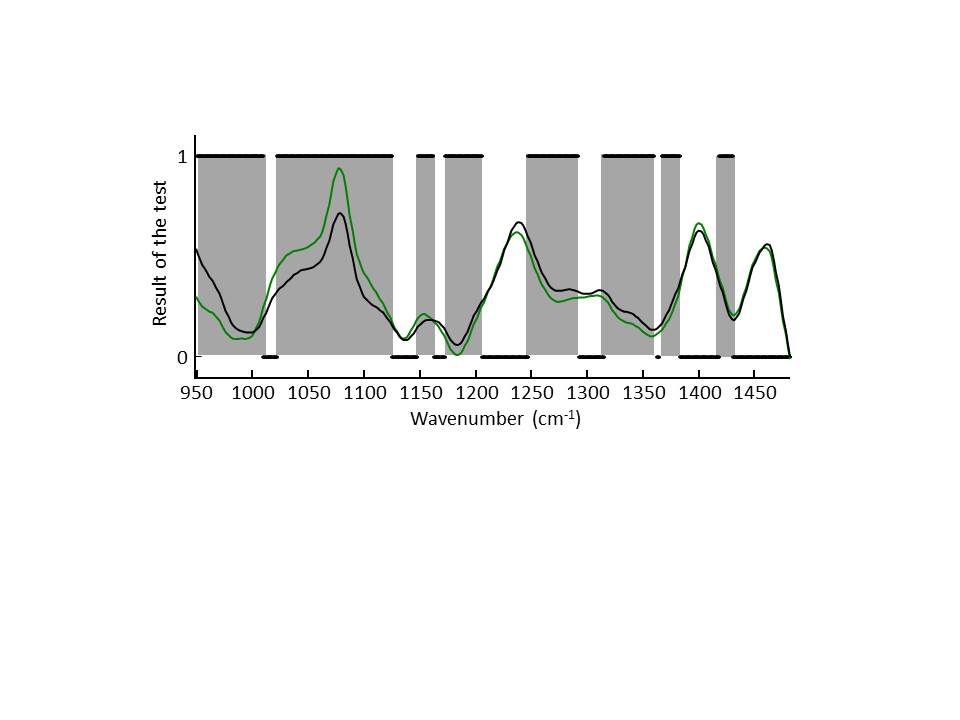 |
| --- |
| **Supplementary Fig. 1** Result of the t-test for the spectra of normal tissue and tumor tissue. The gray areas indicate spectral regions in which the null hypothesis can be rejected. For better classification, the mean spectra of normal tissue (green) and tumor tissue (black) are also shown. A test result of 0 means that the null hypothesis cannot be rejected. A test result of 1 means that the null hypothesis can be rejected; the mean values are different. The spectral regions 995 cm^-1^ – 1005 cm^-1^, 1102 cm^-1^ – 1114 cm^-1^ and 1151 cm^-1^ – 1155 cm^-1^ used by the classification algorithm show significantly different mean values of the extinction values for the two classes according to the t-test. |

| 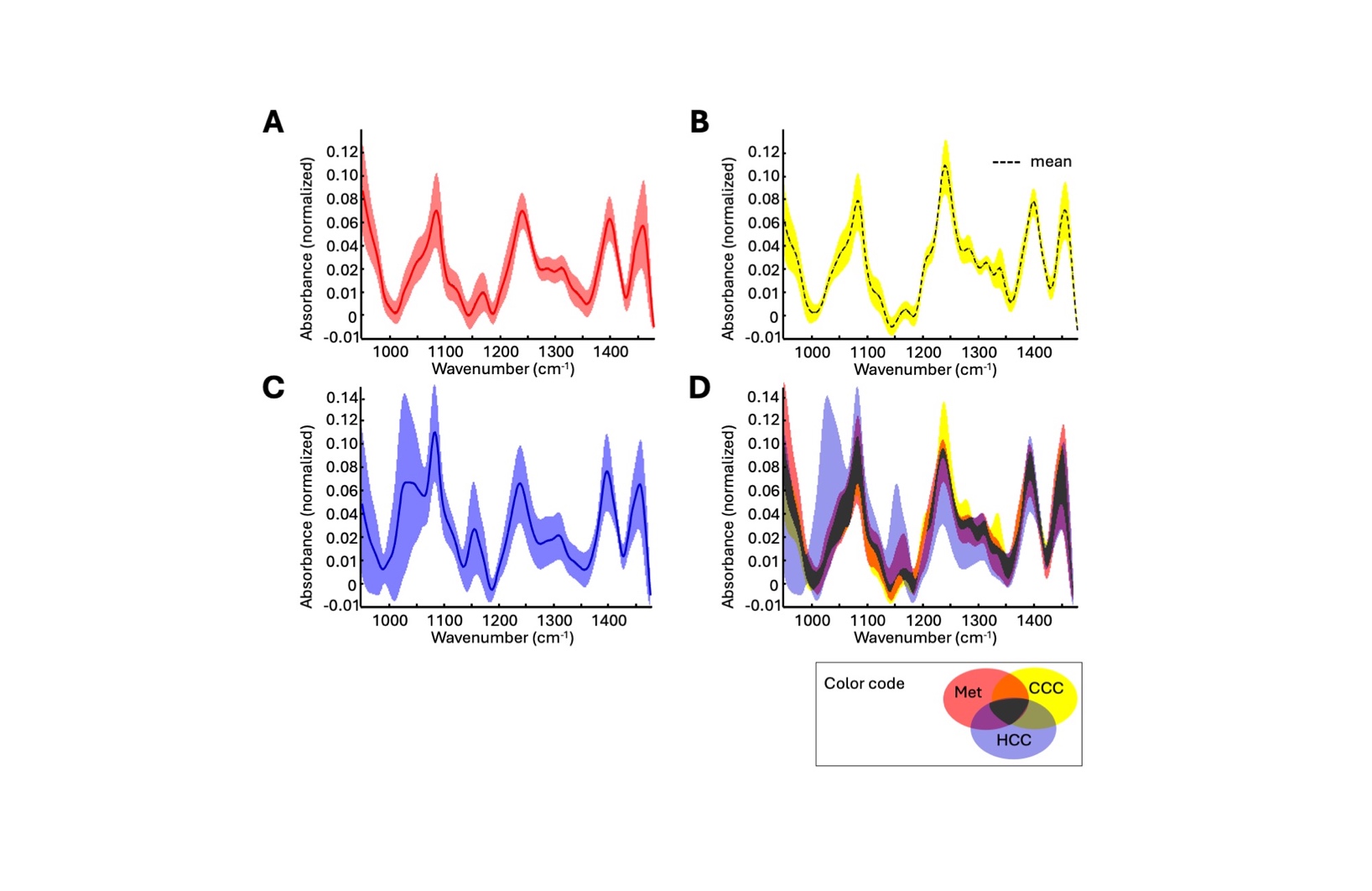 |
| --- |
| **Supplementary Fig. 2** Mean (μ), bold lines, and two-sided standard deviation (±σ) bands of IR ATR spectra recorded from native tissue samples **A)** metastases (Met), **B)** CCC and **C)** HCC tissue. Plot **D)** shows the overlay of the three μ ±σ bands. |

| 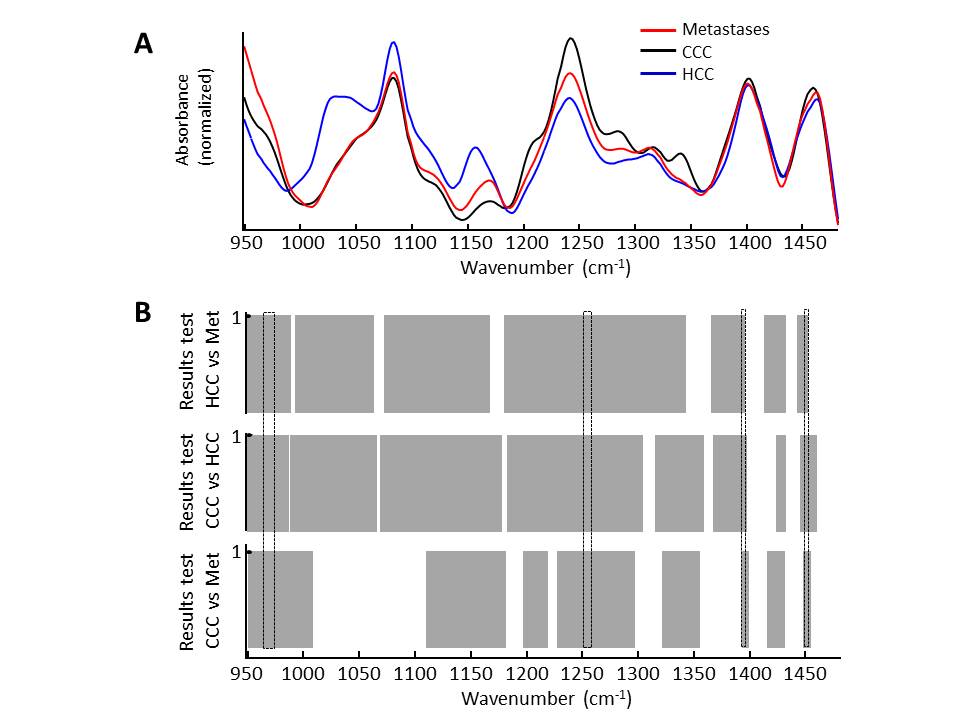 |
| --- |
| **Supplementary Fig. 3** A) Plot of the mean spectra of CCC, HCC, and metastases. B) Results of the pairwise t-tests. The gray areas indicate the spectral regions in which the null hypothesis can be rejected. The dashed areas mark the spectral regions selected by the classification algorithm. The three pairwise t-tests initially show that HCC in particular differs very clearly from CCC and metastases. In contrast, there is greater similarity between CCC and metastases. The test result 0, meaning that the null hypothesis cannot be rejected, occurs significantly more frequently here. The regions selected by the classification algorithm lie in areas where the t-test yields a test result of 1 in each case. |

**Supplementary protocol – Measurement using ATR IR fiber probe**

**1. Detector Cooling**

Cooling Procedure:

- Before starting measurements, cool the MCT detector using liquid nitrogen, following the standard procedure used for other MCT detectors.

Important:

- Add liquid nitrogen slowly to prevent cryogenic blowback. Do not pour too much at once.

Filling:

- Ensure the detector is completely filled with liquid nitrogen.

Signal Check:

- After cooling, verify that the interferogram amplitude is within 23,000–24,000.
- This can be checked via the “Check Signal” tab in the parameters file.

Reference: Example interferogram provided:


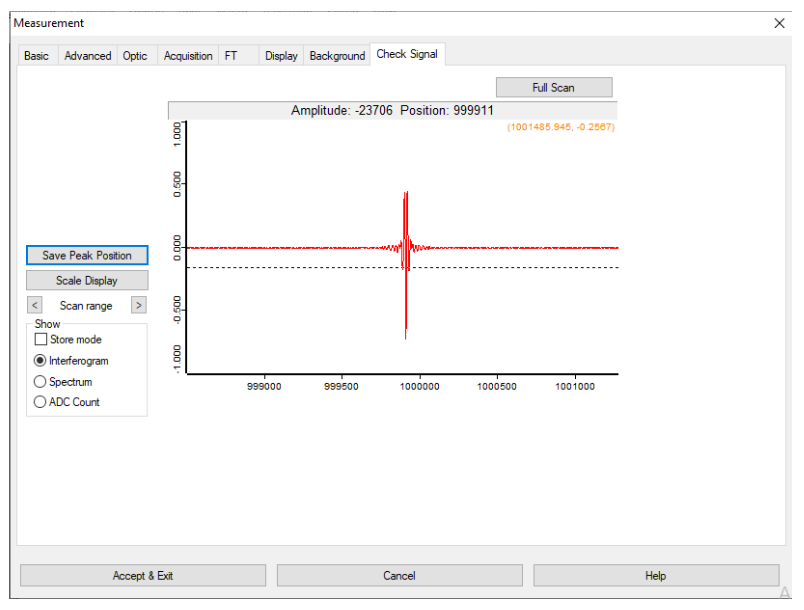


**2. Cleaning the ATR Crystal and Probe**

Cleaning Agents:

- Use distilled water and alcohols (ethanol or isopropyl alcohol).
- Do not use strong acids, alkalies, or other harsh chemicals.

Materials Note:

- The ATR crystal is made of germanium (Ge).
- The probe tip and shaft are made of PEEK.

Cleaning Tools:

- Use soft cotton swabs or paper tissues.
- Be gentle to avoid scratching or damaging the soft Ge crystal.

Protection:

- Always protect the probe head from scratches and breakage.

**3. Background Spectrum Measurement**

Before Each Measurement:

- Record a background spectrum of ambient air.
- Ensure the ATR crystal is clean prior to background measurement.

**4. Measurement Considerations**

Tissue measurement:

- Tissue needs to be measured within 60 mins after being resected
- After receiving a tissue sample, cut the sample within the area of interest and measure the freshly cut surface to reduce drying effects influencing the spectral signals

Probe Immersion:

- If immersing the probe into a solution, do not submerge the junction where the ATR tip attaches to the fiber.
- Submersion of the junction can allow fluid to enter gaps, damaging the fiber ends.

**5. Handling Optical Fibers**

Bending Restrictions:

- Do not bend optical fibers forcefully.
- Maintain a minimum bending radius of 140 mm (bending diameter 280 mm).

Holding the Probe:

- Always hold the probe by the shaft (grey area).
- Never hold or pull on the fiber (black area).
